# Molecular mechanisms, off‐target activities, and clinical potentials of genome editing systems

**DOI:** 10.1002/ctm2.34

**Published:** 2020-05-13

**Authors:** Nannan Zheng, Liyang Li, Xiangdong Wang

**Affiliations:** ^1^ Zhongshan Hospital Institute for Clinical Science Shanghai Institute of Clinical Bioinformatics Shanghai Engineering Research for AI Technology for Cardiopulmonary Diseases Fudan University Shanghai China

**Keywords:** CRISPR/Cas12a, CRISPR/Cas9, off‐target, transcription activator‐like effector nucleases, zinc finger nucleases

## Abstract

Methodologies of genome editing are rapidly developing with the improvement of gene science and technology, mechanism‐based understanding, and urgent needs. In addition to the specificity and efficiency of on‐target sites, one of the most important issues is to find and avoid off‐targets before clinical application of gene editing as a therapy. Various algorithms, modified nucleases, and delivery vectors are developed to localize and minimize off‐target sites. The present review aimed to clarify off‐targets of various genome editing and explore potentials of clinical application by understanding structures, mechanisms, clinical applications, and off‐target activities of genome editing systems, including CRISPR/Cas9, CRISPR/Cas12a, zinc finger nucleases, transcription activator‐like effector nucleases, meganucleases, and recent developments. Current genome editing in cancer therapy mainly targeted immune systems in tumor microenvironment by ex vivo modification of the immune cells in phases I/II of clinical trials. We believe that genome editing will be the critical part of clinical precision medicine strategy and multidisciplinary therapy strategy by integrating gene sequencing, clinical transomics, and single cell biomedicine. There is an urgent need to develop on/off‐target‐specific biomarkers to monitor the efficacy and side‐effects of gene therapy. Thus, the genome editing will be an alternative of clinical therapies for cancer with the rapid development of methodology and an important part of clinical precision medicine strategy.

## Graphical abstract

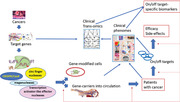

**Abbreviations**
AAV: adeno‐associated virus
ABE: adenine base editor
CAR‐T: chimeric antigen receptors T cells
CBE: cytosine base editor
CRISPR: clustered regularly interspaced short palindromic repeats
DSB: double stranded breaks
dsODN: double‐stranded oligodeoxynucleotides
FnCpf1: Francisella cpf1
gRNA: guide RNA
HDR: homology‐directed repair
NHEJ: non‐homologous end joining
NLS: nuclear localization signal
PAM: protospacer adjacent motif
PE: primer editing
pegRNA: prime editing guide RNA
REC: α‐helical recognition
RNP: ribonucleoprotein
SNV: single nucleotide variants
TALEN: transcription activator‐like effector nuclease
ZFN: zinc finger nucleases

## 1 INTRODUCTION

Genome editing technology has been heavily scrutinized and continuously been refined and explored since the inception, ultimately resulting in various tools, such as meganucleases, zinc finger nucleases (ZFNs), transcription activator‐like effector nuclease (TALENs), clustered regularly interspaced short palindromic repeats (CRISPR) systems, and several novel genome editing tools such as base editors and prime editing. There is a development need of genome editing from biological research to clinical treatments of diseases related to gene abnormalities and gene modification‐based cell therapy.^1^ However, a multitude of issues still plague genome editing technologies, such as off‐target effects, ethical implications, and efficiencies for transferring and editing genes. Among those, off‐target effects are of high concern due to the potential of extensive amounts of unexpected damages to off‐target sites and cellular toxicity. In the clinical applications of gene editing technologies, numerous clinical trials on cancer, infectious diseases, β‐thalassemia, and various inherited disorders are underway. The present review aims at understanding the structures, mechanisms, regulations, and potential clinical applications in cancer of genome editing systems. We furthermore address how off‐targets are generated via gene editing and potential methodologies to detect and decrease the off‐target effects to enable clinical applications in future.

## 2 MEGANUCLEASE

### 2.1 Mechanisms of Meganuclease system

**FIGURE 1:**
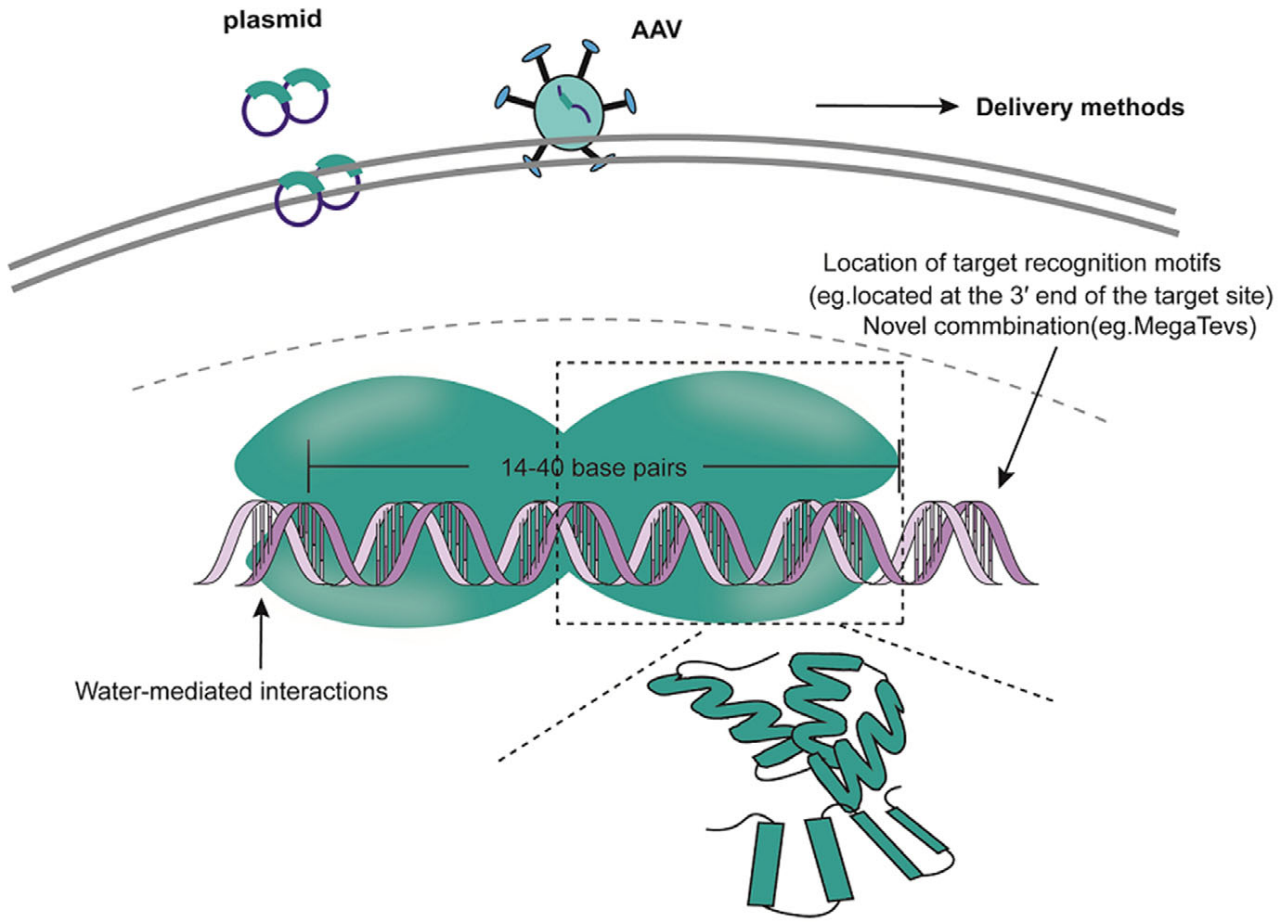
The mechanisms of MEGANUCLEASES system and the influence factors of off‐targets. Each monomer can form αββαββα fold, with four‐stranded antiparallel β‐sheets to recognize and combine with target sequence. Meganucleases identify approximately 14‐40 base pairs in the target sequences. The off‐target activities induced by meganucleases are affected by the structure of meganucleases, and the delivery methods. I‐Crel has the water‐mediated interactions between the target bases and 15 amino acid side chains.

Meganucleases in mitochondria belong to LAGLIDADG family of homing endonucleases, which recognize sites corresponded to intron‐free or intein‐free genes.^2^ They can combine with longer DNA sequences approximately 14‐40 base pairs than restriction enzymes and have two forms, I‐SceI and I‐Crel that are widely used in genome editing^2^ (Figure 1). Each meganuclease protein chain contains one or two conserved LAGLIDAG motifs to take shape as homodimeric proteins and cleave palindromic DNA sequence. Natural meganucleases act as mobile genetic elements, and induce double strand breaks (DSBs) to insert intron in the targeted sites. The I‐SceI was able to cut the bottom and top DNA strand sequentially, resulting in less toxicity and generation of homologous recombination.^3^ But considering that there is limited natural meganucleases to identify various desired sites in practical application, time‐consuming and high‐costing artificial meganucleases are necessary to be engineered, which may limit the extensive use of meganucleases in genome editing.

### 2.2 Off‐target activities of meganuclease

Meganucleases may have the lower rate of off‐targets due to specific sites targeted by spanning long sequences and high cleavage specificity,^4^ while more studies should be conducted to verify its efficacy. The specificity of I‐Crel can be influenced by the water‐mediated interactions between the target bases and ∼15 amino acid side chains or the location of target recognition motifs. The location of target recognition motifs at the 3′ end of the target site could increase the efficiency of on‐targets.^5^ The combination of meganuclease with a TAL array or I‐TevI (a GIY‐YIG enzyme) or the second generation of meganuclease can also reduce off‐target activities, for example, the PCSK9 gene editing in Macaque mulatta kidney cells.^5^, ^6^

**HIGHLIGHTS**

- Genome editing technologies are rapidly developing and become more important in clinical and translational medicine
- Clinical trials of genome editing therapy are on the way to show efficacy and on/off‐targets
- Off‐targets are critical obstacles clinical application of gene editing and must be clarified
- It is necessary to efficiently and dynamically monitor on/off‐targets and efficacy.

## 3 ZINC FINGER NUCLEASES

### 3.1 Mechanisms of ZFNs system

**FIGURE 2:**
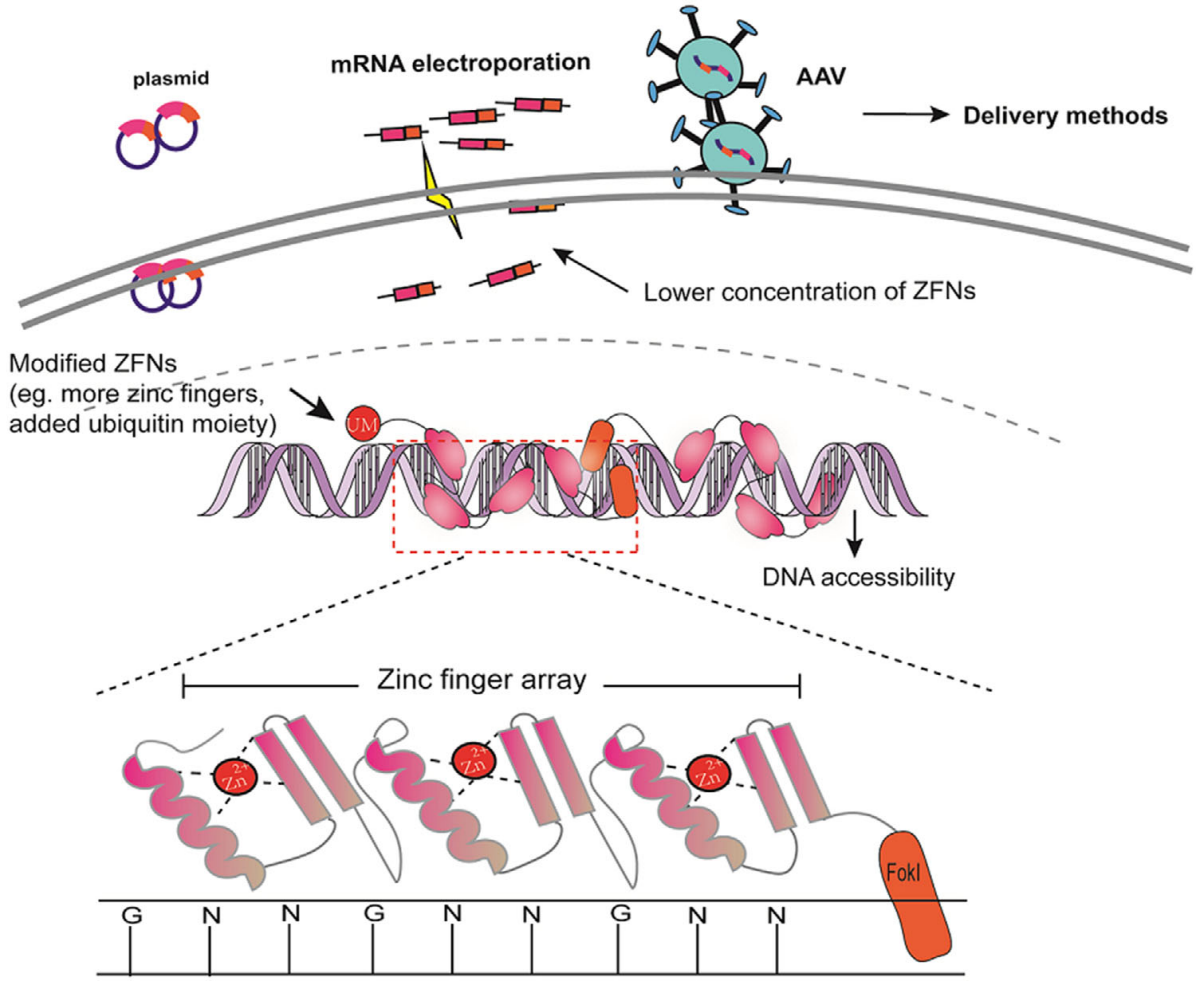
The mechanisms of ZFNs system and the influence factors of off‐targets. ZFNs system is composed of C2H2 zinc fingers formed as conserved ββα supersecondary structure including a Zn^2+^ and FokI C‐terminase with 96 amino acid residues. ZFNs identify the strands as a dimer and every finger can recognize three base pairs, generally GNN. The off‐target activities induced by ZFNs are affected by the concentration and structure of ZFNs, DNA accessibility, and the delivery methods.

**FIGURE 3:**
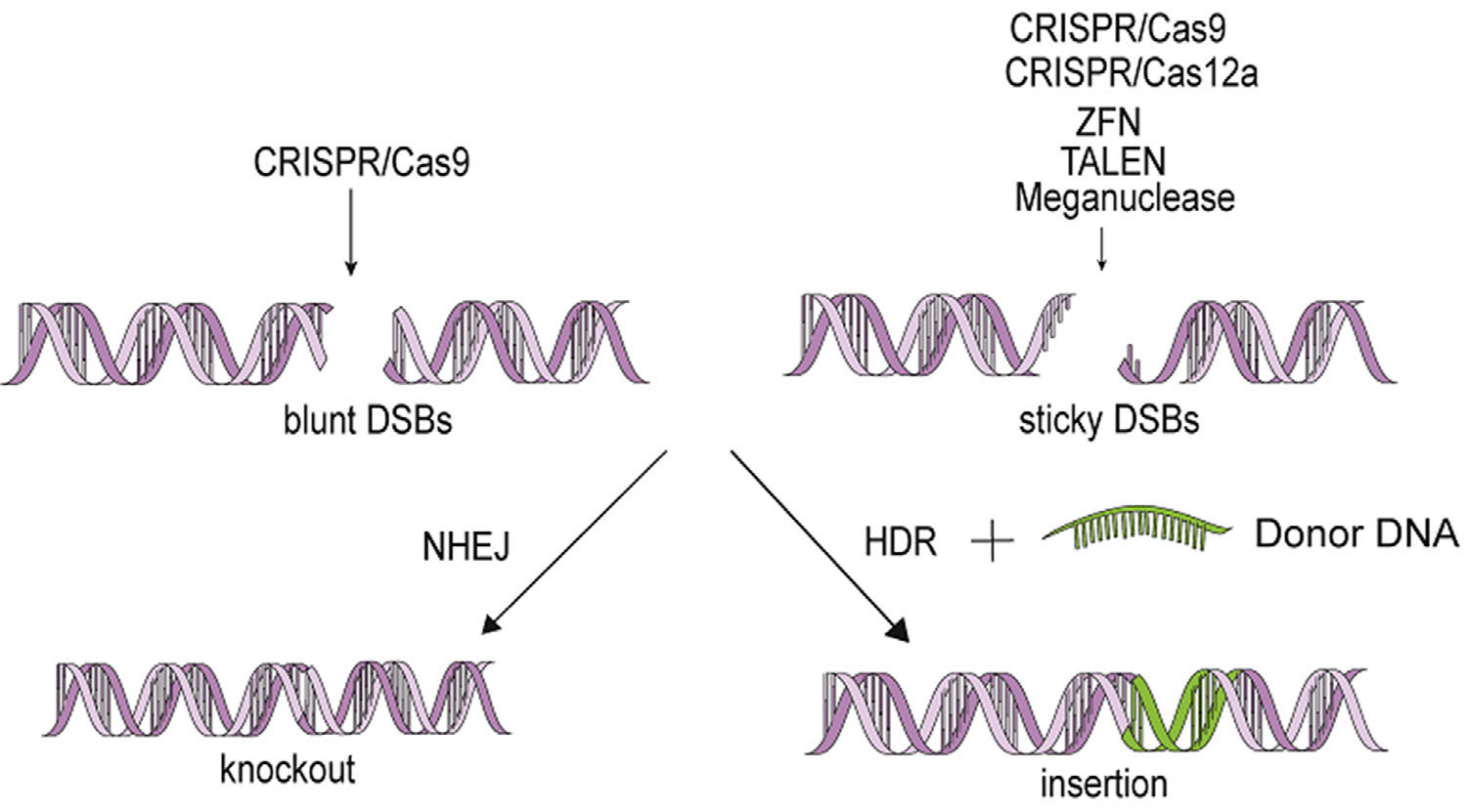
The repair way of genome editing. CRISPR/Cas9 system can induce both blunt and sticky ends according to the Cas9 orthologs, and all the other systems mentioned generate sticky ends after cleavage. Generated DSBs are repaired by NHEJ and HDR. HDR is more precise than NHEJ which leads to more meaningless mutations. In the process of HDR, a donor DNA template is necessary to insert the intended sequence into targeted locus.

ZFNs consist of a DNA‐binding domain and a DNA‐cleavage domain (Figure 2), and act as artificial restriction enzymes and prominent tools in genome editing.^7^ ZFNs‐targeted sites can be extended within a reasonable range via linking three and more zinc fingers, of which each contains about 30 amino acids in a conserved ββα supersecondary structure and recognizes three or four base pairs of DNA.^8^ FokI as a restriction endonuclease in *Flavobacterium okeanokoites* consists of a DNA‐binding domain at N‐terninal residues and a cleavage domain at C‐terminal residues and two FokI C‐terminuses with 96 amino acid residues are needed to constitute the DNA‐cleavage domain of ZFNs and function as dimers when two binding sites are in proximity separated by less than six bps.^8^ In the process of genome editing, ZFNs are introduced into cells by viral or nonviral vectors, which have the ability to enter every genome compartment, even mitochondrial DNA.^9^ During mRNA translation to protein in hematopoietic stem/progenitor cells, more than three zinc fingers are linked together to combine with at least nine base pairs in the major groove of DNA by amino acids on the surface of α‐helix.^10^ Each set of zinc fingers can connect to different specific DNA sequence with GNN and generate stagger ends with a 5′ overhang. DSBs are repaired by non‐homologous end joining (NHEJ) and homology‐directed repair (HDR), as shown in Figure 3. NHEJ is more efficient than HDR due to its activity during the whole cell cycle where HDR is almost absent in G1 phase, and most active in the S phase.^11^ In normal human proliferating cells, NHEJ repairs 75% of DSBs while HDR about 25%,^12^ through simply ligating the two ends, during which a small insertion or deletion (indel) may be created. In HDR, a donor DNA with homology arms is necessary as a template. DSBs repair can make single‐nucleotide changes and the insertion of large and multigene cassettes.^13^ The integrase‐defective lentiviral vectors and single‐stranded oligodeoxnucleotides were used to deliver the homologous donor template, of which adeno‐associated virus (AAV6) triggers higher rate of HDR (35%).^14^

### 3.2 Off‐target activities of ZFNs

Zinc fingers can artificially manipulate the target sites by editing the combined sequences without the unique recognition site like protospacer adjacent motif (PAM) in CRISPR/Cas9 systems. Each tandem array of Cys2His2 zinc fingers can precisely combine with seven of nine sites within the target site, but two positions in ZFPs at C‐terminal finger α‐helix were quietly uncertain.^7^ A number of influencing factors lead to less off‐targets and more efficient outcomes, including additional zinc fingers to improve quality, high specificity of Cys2His2 zinc fingers, or lower concentration of ZFNs.^15^ Strict control of ZFNs half‐life can alleviate cytotoxicity, evidenced by the findings that N‐terminus of ZFNs could combine with a ubiquitin moiety and be inactivated by the small molecule proteasome inhibitor.^7^ As a consequence of off‐targets, DSBs influence cell viability and lead to the lethality of ZFN genome editing.

### 3.3 Clinical potentials of ZFNs in cancer

ZFNs were utilized in acquired immune deficiency syndrome in phase I of clinical trials to reduce human immunodeficiency virus (HIV) load by ZFN‐mediated CCR5‐modified CD4+ T cells, named SB‐728, which was reported to be safe and efficient in the therapeutic effects of 12 patients.^16^ SB‐913, SB‐318, ZFN‐603, and ZFN‐758 have also been registered in Clinicaltrials.gov to enter the clinical trials on several human diseases, including human papillomavirus‐related malignant neoplasm. As to cancer treatment, cancer immunotherapy aims to transport high‐avidity T cell receptor genes separated from tumor‐specific lymphocytes into polyclonal T cells, where ZFNs could inhibit endogenous surface expression of receptors and enhance exogenous ones through disrupting endogenous T cell receptor β‐ and α‐chain genes.^17^ ZFNs target the long terminal repeat of human T‐cell leukemia virus type‐1 and introduce the DSBs in the cells, to reduce the expression of the virus in adult T‐cell leukemia‐derived T cells or eliminate provirus‐positive cells.^18^ However, no results have been reported yet in the phase I clinical trials on the recurrent malignant glioblastoma using ZFN‐mediated genetically edited T cells (GRm13Z40‐2 CTLs) (Clinicaltrials.gov, NCT01082926) and the application of ZFN‐603 and ZFN‐758 in the treatment of human papillomavirus‐related malignant neoplasm (Clinicaltrials.gov, NCT02800369).

## 4 TRANSCRIPTION ACTIVATOR‐LIKE EFFECTOR NUCLEASES

### 4.1 Mechanisms of TALENs system

**FIGURE 4:**
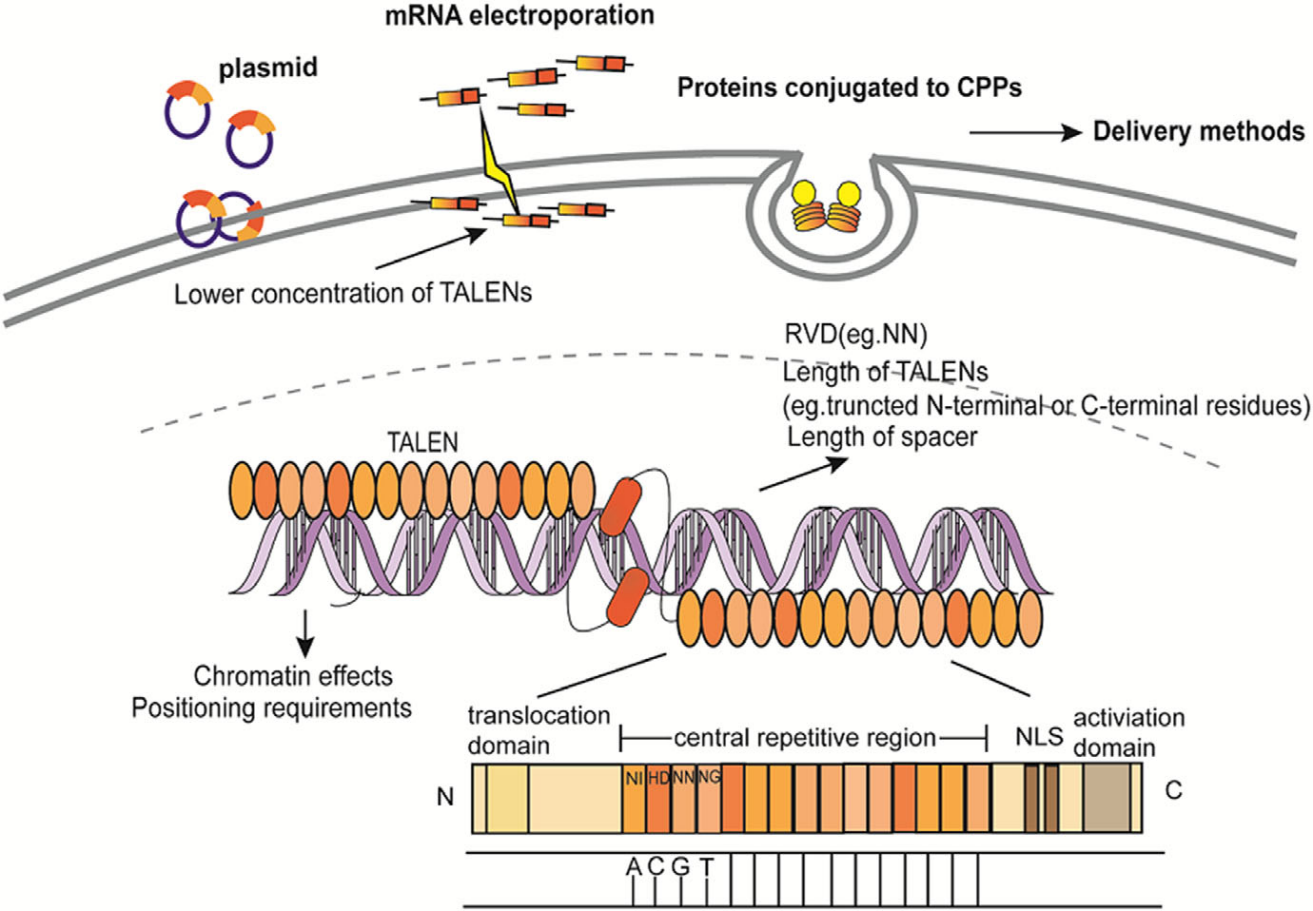
The mechanisms of TALENs system and the influence factors of off‐targets. TALENs system is composed of more than 11 monomeric TAL effectors and FokI. Each TAL effector contains an N‐terminal translocation domain, a nuclear localization signal (NLS), a transcription activation domain, and a central repetitive region with 13‐29 tandem repeat units. TALENs identify the target sequences as a dimer and the binding sites are separated by 17 bases. The desired DNA sequences are identified on basis of one repeat‐to‐one base principle, with hypervariable residues NN, NI, HD, and NG recognizing G, A, C, and T, respectively. The off‐target activities are affected by the concentration and structure of TALENs, the chromatin effects, and the delivery methods.

Designed TALENs have higher efficiency and lower off‐target effects than ZFNs,^19^ including a DNA‐cleavage domain of FokI and a DNA‐binding domain (Figure 4). DNA‐binding domain contains a translocation domain at N‐terminal residues, a nuclear localization signal (NLS) that is necessary to match DNA targets, a transcription activation domain, and a central repetitive region with 13‐29 tandem repeat units named TAL effectors. Each TAL effector has 34 amino acids and two variable amino acids at position 12 and 13. The amino acid at position 13 functions to identify specific nucleotides, while the one at position 12 stabilizes the repeat variable di‐residues.^20^ TALENs are introduced into the cells by either being conjugated to cell‐penetrating peptides, co‐transfecting two plasmids encoding a pair of TALENs, or injecting mRNA into mice directly.^21^ The desired DNA sequences are identified on basis of one repeat unit‐to‐one base principle, where almost all engineered TALEN repeat arrays recognize G, A, C, and T, through four domains with hypervariable residues NN, NI, HD, and NG, respectively.^22^ TALENs correctly identify the specific long DNA sequences, which is affected by the number of CpG dinucleotides around the target sites.^23^ After the cleavage domains trigger the DSBs with 5′ overhangs, NHEJ and HDR repair the truncation and HDR accounts for up to 20%.^24^

### 4.2 Off‐target activities of TALENs

TALENs induced relatively less off‐target effects and cytotoxicity, for example, 2.4% off‐target for targeting human β‐globin gene in pluripotent stem cells,^25^ or about 10% off‐target sites in human pluripotent cells by SELEX,^26^ and no detection in animal models.^27^ Repeat variable di‐residues combine with specific DNA preferentially rather than only one since NN could identify both A and G. Truncating N‐terminal and C‐ terminal domains with 136 and 63 residues were more efficient and the optimal length and concentration of TALENs also influence the off‐targets.^28^ Specially designed TALENs can increase threefold of TALEN monomer activity in cells.^29^

### 4.3 Clinical potentials of TALENs in cancer

As a promising HIV therapy, TALENs are more precise and flexible than ZFNs, and induce fewer off‐target sites, although there are still obstacles to be faced, for example, time consumption and difficulty to construct.^30^ In large animal models, *Pdx‐1* knockout pigs or human α‐lactalbumin genes introduced goat were constructed via TALENs.^31^, ^32^ TALENs were experimentally applied for cancer therapy, to produce t(11;22)(q24;q12) and t(2;5)(p23;q35) translocations in human cells,^33^ target E7 gene of HPV in SiHa cells for cervical cancer,^34^ or modify exon 3 of the HPRT1 in human myeloma cells.^35^ TALENs have also entered the stage of clinical trails in cancer therapy. T27, T512 (Clinicaltrials.gov, NCT03226470), and TALEN‐HPV16 E6/E7 or TALEN‐HPV18 E6/E7 (Clinicaltrials.gov, NCT03057912) were registered to treat the cervical intraepithelial neoplasia. UCART22 (Clinicaltrials.gov, NCT04150497), UCART123 (Clinicaltrials.gov, NCT03190278) and UCARTCS1A (Clinicaltrials.gov, NCT04142619) are all undergoing the phase I clinical trials of hematological malignancies.

## 5 CRISPR/Cas9

### 5.1 Mechanism of CRISPR/Cas9 system

**FIGURE 5:**
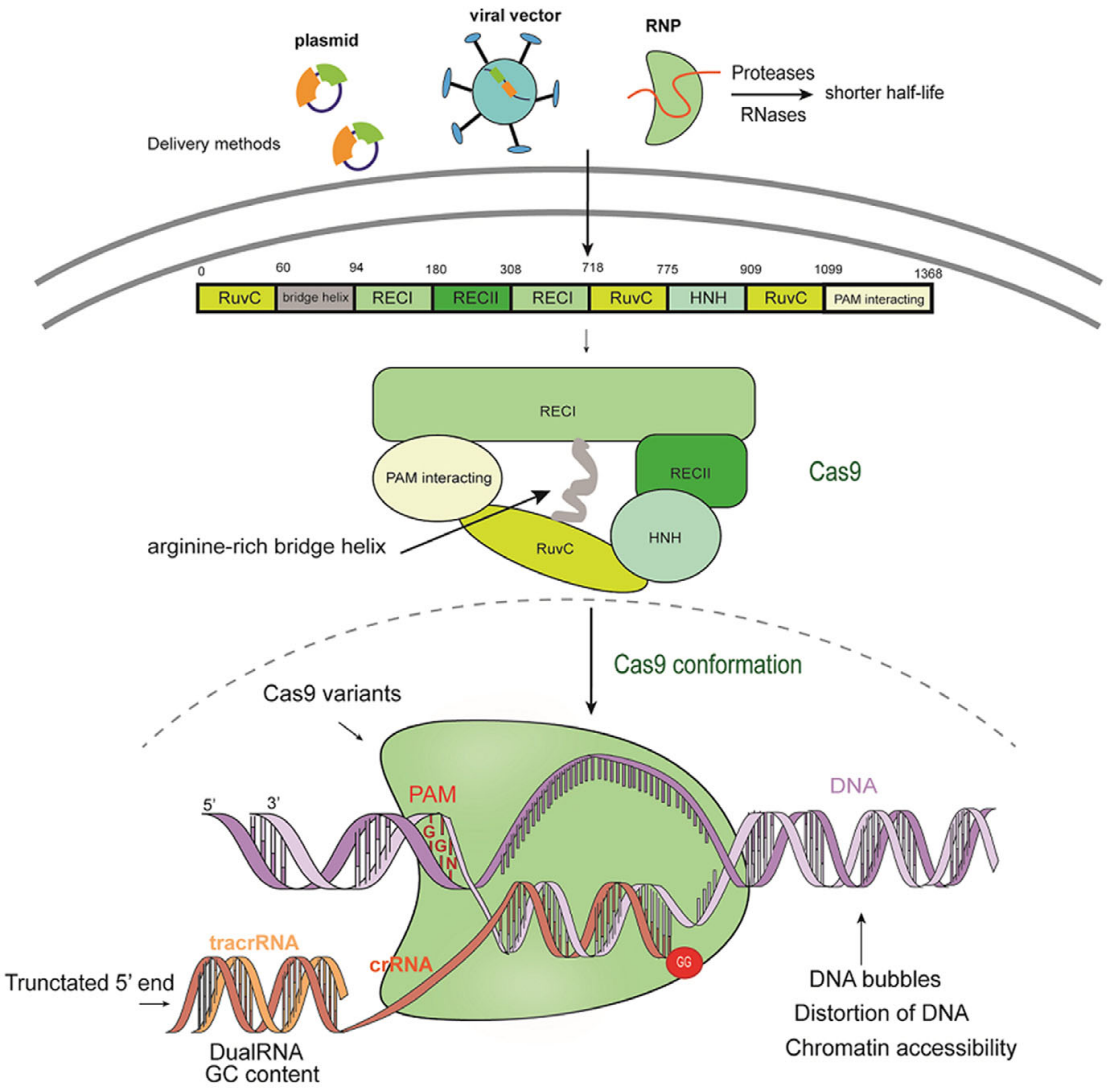
The mechanisms of CRISPR/Cas9 system and the influence factors of off‐targets. CRISPR/Cas9 system is composed of Cas9 and a gRNA. Cas9‐gRNA complex identifies the strand including a PAM (NGG) at the 3′ end adjacent to the 20‐base pair target site. Cas9 consists of a α‐helical recognition (REC) lobe and nuclease lobe containing RuvC and HNH domain, which are joined by a bridge helix and cleave the two DNA strands simultaneously after conformation. The off‐target activities induced by CRISPR/Cas9 are affected by the non‐canonical PAM at off‐target sites, DNA accessibility, the structure of gRNA and Cas9 as well as the delivery methods of the Cas9‐gRNA compound.

CRISPR is a form of adaptive immunity in approximately 87‐90% archaeon and 40‐45% of bacteria.^36^ CRISPR systems play an important role in genome editing as well as RNA and base editing.^37^ CRISPR/Cas9 system includes Cas9 proteins (such as canonical *Streptococcus pyogenes* Cas9 [SpCas9], *Campylobacter jejuni* Cas9 [CjCas9], *Staphylococcus aureus* Cas9 [SaCas9], *Neisseria meningitides* [NmCas9]), a specificity‐determining CRISPR RNA (crRNA), and an auxiliary trans‐activating RNA (tracrRNA)^38^ (Figure 5). crRNA and tracrRNA are transformed to a dualRNA or single‐guide RNA (sgRNA). Cas9 is composed of the recognition lobe and the nuclease lobe including RuvC and HNH domains, which are joined with a highly conserved arginine‐rich helix, to form contacts with the sgRNA.^39^ The Cas9‐sgRNA complex identifies the strands, including a PAM, typically NGG, and among targeted 20 base pairs in upstream of PAM, eight to 12 bps are pivotal for recognition and the cutting site is at the third base pairs upstream the PAM.^40^ HNH conformations are subsequently activated and communicated with the α‐helix acts as a switch through the RuvC domain to cleave the two DNA strands simultaneously, inducing double stranded breaks (DSBs) at target sites.^39^ DSBs were also repaired by NHEJ or HDR, during which the rate of HDR is lower than that in ZFNs.^41^

### 5.2 Off‐target activities in CRISPR/Cas9

CRISPR/Cas9 may cause more than 50% frequency of off‐target activity, leading to the undesired DNA damage and cytotoxicity.^42^, ^43^, ^44^, ^45^, ^46^ The comparative study between CRISPR/Cas9 and TALEN showed that CRISPR/Cas9 has higher efficiency and off‐targets in the generation of a *Mstn*‐knockout lamb.^47^ Recent studies on large animal models like sheep and goat have shown that few off‐target activities are detected, contrary to in vitro experiments.^48^, ^49^, ^50^ Off‐target sites contain noncanonical PAMs and several different nucleotides from on‐target sites.^43^ The 5′‐end of gRNAs is better tolerated to mismatches than the 3’‐end.^46^ One or more mismatches in the seed region can block the activation of Cas9, while three or more mismatches result in the binding of DNA sequences to the Cas9 L2 loop, hindering HNH conformation and preventing cleavage.^51^ The structures and compositions of gRNAs in the system play a key role in off‐target activities. Structurally, dualRNA is more efficient in discriminating off‐target sites compared to sgRNA, since the shorter sgRNA construct is less tolerant to off‐target mutations than the longer and more‐active one.^52^ A dual‐guide RNA (dgRNA) with particular nucleotide changes in tracrRNA could significantly improve Cas9 RNP activity.^53^ Experimental data showed that truncating more than three nucleotides at the 5′‐end of the gRNA could increase the specificity and high‐GC content of gRNA can increase off‐targets via stabilizing the gRNA‐DNA hybridization at unintended sites.^54^

In order to reduce off‐targets, gRNAs were modified by incorporating next‐generation bridged nucleic acids, nucleic acids at specific sites were locked, or the GG motif was designed at the 3′‐end of target sequences.^55^ Some Cas9 variants were engineered to mitigate off‐target effects such as sniper‐Cas9,^56^ HypaCas9,^57^ eSpCas9,^58^ and so on, while on‐target efficiency might be reduced simultaneously. The chromatin accessibility and delivery methods of Cas9‐gRNA into cells also influence Cas9 binding specificity,^59^ including plasmid DNA vectors, viral vectors, or Cas9‐gRNA ribonucleoprotein (RNP) complexes. Compared to plasmid or viral vectors, RNP initially presents with a high concentration, but has a shorter half‐life due to the degradation by endogenous proteases and RNases to lower the off‐target effects and maintain the on‐target cleavage efficiency.^60^

### 5.3 Clinical potentials of CRISPR/Cas9 in cancer

CRISPR/Cas9 was suggested as a significant potential for clinical application, for example, in inherited genetic diseases,^61^ infectious diseases,^62^ cancer,^63^ and so on. This system has been mostly applied for the creation of animal models for human diseases,^64^ while there are few large animal models or clinical trials on cancer therapy via CRISPR/Cas‐mediated genome editing. T cells were modified by CRISPR/Cas9 to generate more potent chimeric antigen receptors T cells (CAR‐Ts), which was a prospective way to cure cancer.^65^ CRISPR/Cas9 was used to target exon 3 genomic loci to ablate CD33 expression in CAR‐Ts as an antigen‐directed immunotherapy for acute myeloid leukemia.^66^ Tumor progression was suppressed in murine ovarian cancer cells through PD‐L1 disrupted by CRISPR/Cas9.^67^ In addition, CRISPR/Cas9 systems can interfere with microRNAs and long noncoding RNAs, to play an important role in cancer.^68^ However, high off‐target mutations and unpredictable side effects of the system are still considered before clinical trials.^69^ The in vitro studies demonstrated that CRISPR/Cas9‐mediated DSBs could activate p53‐mediated DNA damage response, to inhibit the efficiency of editing and induce the selection against the cells with functional p53 pathway.^70^, ^71^ Mutations potentially generated from off‐target could endow mutated cells with a carcinogenic “hit” in the clinical process of gene‐edited cells, resulting in a long replicative lifespan and neoplastic changes with time. No alterations of p53 expression in tissues of genetically edited goats can be considered as the evidence of the safety in the clinical application of CRISPR/Cas9.^50^ The human genetic variation may alter on‐ and off‐target specificity at therapeutically intended loci and predispose patients to personal adverse outcomes. More studies on genetically edited large animals are necessary to ensure the clinical safety. As to clinical application, several phase I/II clinical trials are undergoing, including CTX120 in relapsed or refractory multiple myeloma (Clinicaltrials.gov, NCT04244656), anti‐mesothelin CAR‐T cells modified by CRISPR/Cas9 in multiple solid tumors (Clinicaltrials.gov, NCT03545815), CTX110 in B‐cell malignancies (Clinicaltrials.gov, NCT04035434), CRISPR/Cas9 gene‐editing CAR‐T cells targeting CD19 (UCART019) in CD19^+^ leukemia and lymphoma (Clinicaltrials.gov, NCT03166878). In addition, PD‐1 engineered T cells modified by CRISPR/Cas9 were also applied to treat esophageal cancer (ClinicalTrials.gov, NCT03545815), although results were undisclosed.

## 6 CRISPR/Cas12a

### 6.1 Mechanism of CRISPR/Cas12a system

Cas12a is a type V CRISPR effector belonging to class II CRISPR systems for genome engineering.^72^ As compared with CRISPR/Cas9, CRISPR/Cas12a systems has smaller size and its crRNA reduces errors in the synthesis of the nucleotides, particularly when delivered using AAV‐based vectors.^73^ CRISPR/Cas12a can program two or more targets to edit multiplex genes via one plasmid, interrogating gene functions in regulatory networks.^74^ CRISPR/Cas12a is considered to have the lower risk of off‐targets,^75^ although Cas12a is not frequently utilized due to insufficient indel efficiencies. In the genome engineering of maize, over 90% of Cas9‐edited plants had indel mutations, while 0‐60% of Cas12a ‐edited plants had on‐target mutations.^76^ CRISPR/Cas12a needs further improvement for editing efficiency.

**FIGURE 6:**
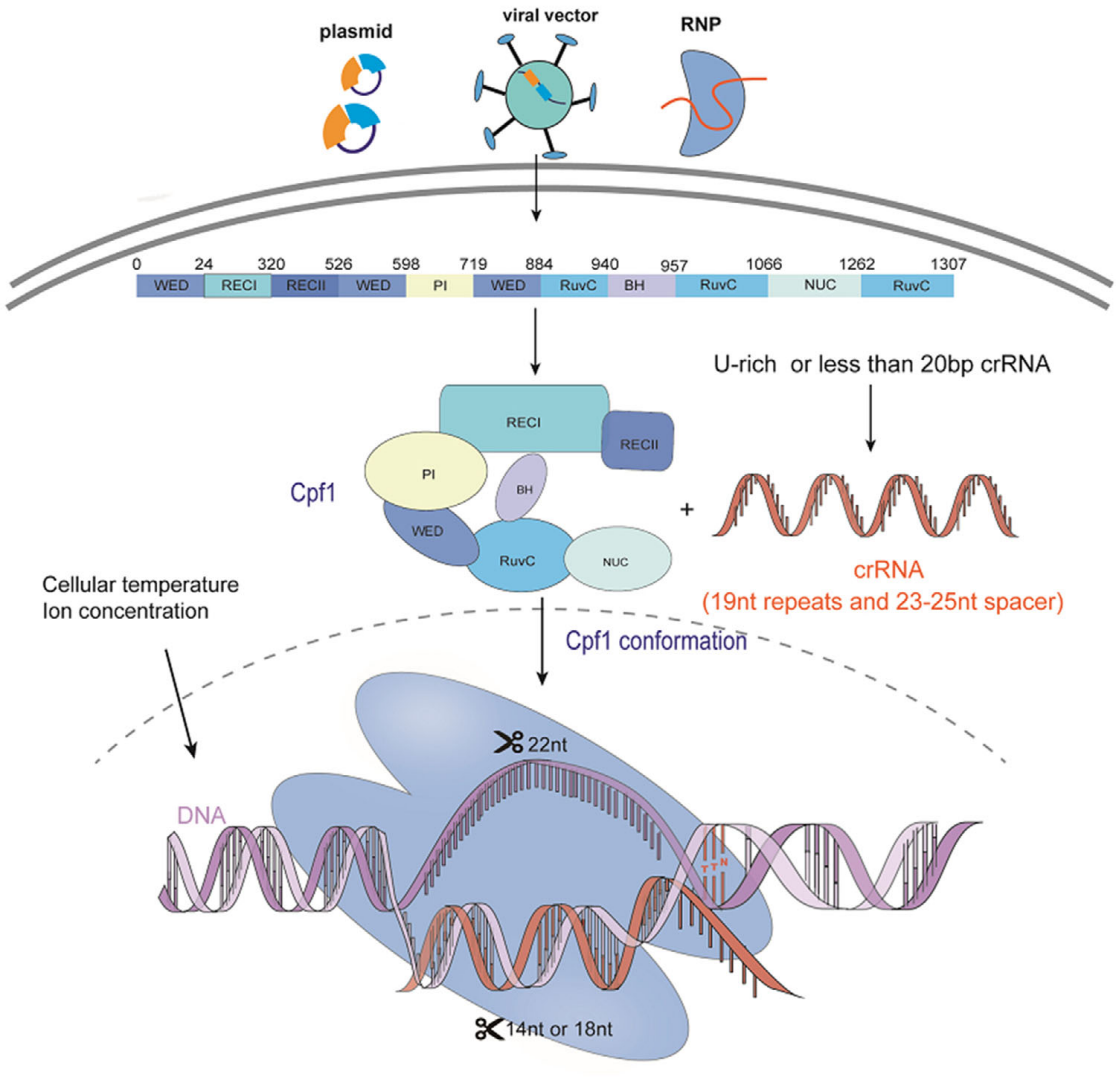
The mechanisms of CRISPR/Cas12a system and the influence factors of off‐targets. CRISPR/Cas12a system is composed of Cas12a and a crRNA. Cas12a‐crRNA complex identifies the strands including a PAM (TTN) at the 5′ end adjacent to the non‐target strand. Mature crRNA in Cas12a has 42‐44 nt in length, including 19 nt direct repeats and a 23‐25 nt spacer sequence. When the spacer sequence is greater than or equal to 20 nt, Cas12a tends to cut 18 bits of non‐complementary chain, while when it is less than 20, Cas12a tends to cut 14 bits. Cas12a includes RuvC and endonuclease domain. Its loose structure can be contracted by the combination with crRNA and cleave the DNA sequences. The off‐target activities induced by CRISPR/Cas12a are affected by the noncanonical PAM at off‐target sites, DNA accessibility, the structure of crRNA and Cas12a, the delivery methods of the Cas12a‐crRNA compound and the cellular environment including the temperature and ion concentration.

In contrast to Cas9, the CRISPR/Cas12a system contains crRNA and lacks tracrRNA (Figure 6). Mature crRNA in Cas12a has 42‐44 nt in length, 19 nt direct repeats, and a 23‐25 nt spacer sequence as a sole stem loop.^77^ When the spacer sequence is greater than or equal to 20 nt, Cas12a tends to cut 18 bits of noncomplementary chain and 14 bits if less than 20 nt.^78^ Chemical modification or 5′ extension of the crRNA can enhance the efficiency of Cas12a.^79^ crRNA identifies the specific sites through the recognition of the PAM sequence with T‐rich not G‐rich, typically 5′‐TTN. Cas12a cleaves a target DNA containing PAM motif on the 5′‐end of the nontarget strand, while the Cas9 on the 3′ end of the nontarget strand.^80^ Cas12a has a RuvC‐like endonuclease domain excising two strands of DNA in a dimeric form.^81^ The putative novel nuclease domain also plays a role in this process.^82^ *Francisella* Cas12a is capable of cleaving both supercoiled and liner DNA. Instead of blunt ends caused by Cas9, Cas12a cleaves two strands of DNA and generates sticky ends with a 4 or 5 nt 5′ overhang far away from the PAM, for NHEJ‐mediated HDR to correct genomic sequences.

### 6.2 Off‐target activities of CRISPR/Cas12a

Cas12a is more specific than Cas9 because the gRNA should be more specific and matching more nucleotides to the complementary DNA sequence.^83^ In the CRISPR/Cas12a‐mediated genome editing in mouse models, the estimated number of off‐target sites was lower than in SpCas9, and off‐targets were not detected at potential sites with two to four bp mismatches.^84^ The major concern is the mismatch between crRNA and target DNA sequences. The structure of crRNA and targeted DNA sequences or delivery methods can affect the specificity of the system. The shorter length of spacer in crRNA, especially less than 20 nt or a U‐rich crRNA, could enhance the specificity of Cas12a cleavage.^85^ As for delivery, RNPs with Cas12a is a valid way to lower the rate of off‐targets as CRISPR/Cas9 system. The cellular environment can also lead to nontarget DNA cleavage. Proper Mn^2+^ concentration along with the appropriate Cas9/Cas12a orthologues can induce RNA‐independent, nonspecific DNA cleavage activities, which could have significant impacts on their applications.^86^

### 6.3 Clinical potentials of CRISPR/Cas12a in cancer

The CRISPR/Cas12a system has great clinical potentials due to smaller molecular sizes and sticky ends of DSBs. For example, CRISPR/Cas12a was applied to correct mutations in Duchenne muscular dystrophy of human cardiomyocytes and the animal disease model.^87^ The editing efficacy and specificity of CRISPR/Cas12a in BRAF‐V600E mutation were carried out to induce specific disruption at a frequently reported driver mutation in various cancers.^88^ More clinical trials are needed to prove CRISPR/Cas12a system as an important tool in the genome engineering.

## 7 OTHER GENOME EDITING SYSTEMS

New tools for genome engineering will be discovered continuously to improve the quality and precision of on‐target. Independent of NHEJ and HDR, base editors, a fusion of the CRISPR/Cas enzyme and deaminase, can induce targeted mutations in genomic DNA and reduce off‐target mutations and DNA damage.^37^ The cytosine base editors BE3, as a fusion of APOBEC1, uracil glycosylase inhibitor, and Cas9‐D10A nickase mutant, can convert C‐G into A‐T base pair, which is a widely used base editor.^89^ The adenine base editor (ABE) with tRNA specific adenosine deaminase and a Cas9 nickase can convert A‐T to C‐G, to inactivate genes by converting four codons into STOP codons and have fewer off‐target mutations than Cas9.^90^ In addition, Komor *et al* engineered CDA1‐BE3 and AID‐BE3 and further developed BE4, SaBE4, BE4‐Gam, and SaBE4‐Gam to further improve the efficiency and reduce the undesired by‐products.^91^ It is possible that base editors can be applied to target single nucleotide mutations in cancer, through inducing precise point mutations in human cells. However, base editors minimize the editing of by‐products such as indels, translocations, or DNA rearrangements, while still indispensably induce off‐targets including DNA and RNA mutations. For example, BE3 can induce single nucleotide variants, mostly C to T conversation.^92^ BE3 and ABE7.10 induced many off‐target RNA single nucleotide variants (SNVs), which could be partly eliminated by modified deaminases.^93^ In the sheep models, obtained lambs with a p.96R > C substitution in SOCS2 by BE3 showed efficiency as 25% and no off‐targets were detected in the edited animals.^49^ Another BE3‐mediated animal model with nonsense codon introgression into caprine FGF5 also showed low off‐target mutations.^94^ BE3 was also reported to successfully introduce nucleotide mutations in pig models.^95^ Those provide the foundation for the clinical application of base editors.

A CRISPR‐associated transposase, composed of Tn7‐like transposase and type V‐K CRISPR effector, can insert segments of DNA 60‐66 bp downstream of the PAM.^96^ Another transposon‐encoded CRISPR/Cas system named INTEGRATE induces the site‐specific DNA integration via Tn7‐like transposon and TniQ.^97^ Different from the canonical genome engineering based on the NHEJ and HDR, transposon‐encoded CRISPR/Cas system can more efficiently insert DNA fragments even in nonmitotic cells, with great potentials of clinical applications.

Prime editing (PE) mainly includes an engineered Cas9 and a prime editing guide RNA called pegRNA that included the target site and desired edit site.^98^ Three generations of PE with increased efficiency were constructed in more than 175 edits in human cells and mouse cortical neurons, showing that prime editing had fewer off‐targets than HDR without reducing the editing efficiency.^98^ Such genome editing system could induce base insertion, deletion, or conservations without DSBs or donor template and target genes at sites ranging from 3‐bp upstream to 29‐bp downstream of the PAM. However, the large size of the engineered Cas9 would be difficult to be delivered, limiting the further application.^99^

## 8 METHODS OF OFF‐TARGET DETECTION

Several methods to detect off‐target effects were developed, including T7 endonuclease I, genome‐wide unbiased identification of DSBs enabled by sequencing (GUIDE‐Seq), in vitro Cas9‐digested whole‐genome sequencing (Digenome‐seq), chromatin immunoprecipitation sequencing (ChIP‐seq), high‐throughput gene translocation sequencing (HTGTS), integrase‐defective lentiviral vector (IDLV), direct in situ breaks labeling, enrichment on streptavidin and next‐generation sequencing (BLESS), circularization for in vitro reporting of cleavage effects by sequencing (CICRLE‐seq), selective enrichment and identification of adapter‐tagged DNA ends by sequencing (SITE‐seq), endonuclease V sequencing (EndoV‐seq), breaks labeling in situ and sequencing (BLISS), and discovery of in situ Cas off‐targets and verification by sequencing (DISCOVER‐Seq). Some were summarized in many reviews,^100^ and here, we more focus on the new detection methods developed in recent 3 years.

Tsai *et al* developed and compared CIRCLE‐seq with GUIDE‐seq, Digenome‐seq, and HTGTS, and found that CIRCLE‐seq was more sensitive and sequencing‐efficient for detecting genome‐wide off‐target cleavage sites of CRISPR/Cas9 systems in vitro.^101^ Detecting off‐target sites without a reference genome might be useful in the organisms without full genomic sequences. BLISS could directly label and quantify the DSBs in situ through unique molecular identifiers with the applicability to low‐input sample.^75^ SITE‐seq, using Cas9 programmed with sgRNAs, was independent on cellular events like DNA repair to detect cut sites in pure genomic DNA and produce sequencing libraries highly enriched for sgRNP cleavage fragments to ensure the specificity.^102^ In EndoV‐seq, endonuclease V‐nicked inosine‐containing DNA strand of genomic DNA was deaminated by ABE7.10, more specific in efficacy and less in off‐targets than CRISPR/Cas9 system.^90^ DISCOVER‐seq was developed on the basis of ChIP‐seq to detect off‐targets in CRISPR systems in vivo by tracking the recruitment of MRE11 at the cut sites in human induced pluripotent stem cells and provided the potential to detect off‐targets in patients with genome editing.^103^ There are more detection methods reported recently, including iBLESS (an improvement of BLESS), qDSB‐seq (an advanced DSB‐seq), target‐enriched GUIDE‐seq (TEG‐Seq), iGUIDE‐seq (an improved GUIDE‐seq), and DIG‐seq (based on Digenome‐seq). The improvement and development of novel methods can provide more possibilities to detect and reduce off‐targets in genome editing systems.

## 9 CONCLUSION AND PROSPECTIVES

Methodologies of genome editing are rapidly developing with the improvement of gene science and technology, mechanism‐based understanding, and urgent needs. Of those, the optimization is on the way when strengths and weakness can be clarified as exampled in Table 1. In addition to the specificity and efficiency of on‐target sites, one of the most important issues is to find and avoid off‐targets before clinical application of gene editing as a therapy. Currently, genome editing in cancer therapy are targeting immune systems in tumor microenvironment by ex vivo modification of the immune cells in phases I/II of clinical trials. We believe that genome editing will be the critical part of clinical precision medicine strategy and multidisciplinary therapy strategy by integrating gene sequencing, clinical transomics and single cell biomedicine.^104^, ^105^, ^106^, ^107^ There is an urgent need to develop on/off‐target‐specific biomarkers to monitor the efficacy and side‐effects of gene therapy, which should be easily and dynamically detected with the clear specificity of diseases, stages, severities, and prognoses.^108^, ^109^, ^110^ Thus, the genome editing will be an alternative of clinical therapies for cancer with the rapid development of methodology and an important part of clinical precision medicine strategy.

**TABLE 1:** The characteristics of genome editing systems and their clinical application in cancer

	Genome editing systems	Composition	Target sequence	DNA repair way	Advantages		Disadvantages	Clinical application in cancer
Nucleases	Meganucleases	Each monomer can form αββαββα fold, with four‐stranded antiparallel β‐sheets	Intron/intein‐free sites	NHEJ/HDR	Fewer off‐targets		Difficult to construct	NA
	ZFNs	C_2_H_2_ zinc fingers and FokI C‐terminuse	Each Zinc finger recognizes three or four base pairs, generally 5′‐GNN‐3′					GRm13Z40‐2 CTL modified by ZFN in recurrent malignant glioblastoma; ZFN‐603 and ZFN‐758 in human papillomavirus‐related malignant neoplasm
	TALENs	A non‐specific DNA‐cleavage domain of FokI and a DNA‐binding domain	Hypervariable residues NN, NI, HD and NG recognizing G, A, C, and T, respectively					T27, T512,TALEN‐HPV16 E6/E7, TALEN‐HPV18 E6/E7 in cervical intraepithelial neoplasia; UCART22, UCART123, UCARTCS1A in hematological malignancies
CRISPR systems	CRISPR/Cas9	Cas9 proteins,a specificity‐determining CRISPR RNA (crRNA), and an auxiliary trans‐activating RNA (tracrRNA)	5′‐NGG‐3′ PAM		Easy to construct	Higher‐efficiency compared with TALEN and Cas12a	More off‐targets than Cas12a; p53 activation	CTX120 in multiple myeloma; anti‐mesothelin CAR‐T cells modified by CRISPR/Cas9 in solid tumors; CTX110 in B‐cell malignancies; UCART019 in CD19+ leukemia and lymphoma; PD‐1 knockout T cells modified by CRISPR/Cas9 in esophageal cancer
	CRISPR/Cas12a	Cas12a protein and crRNA	5′‐TTN‐3′ PAM			Easy to construct; smaller molecular sizes; fewer off‐targets than CRISPR/Cas9	Lower editing efficiency than Cas9	NA
Novel gennome editing tools	Base editors	BE3: rat APOBEC1 and Cas9‐D10A nickase; ABE: tRNA specific adenosine deaminase and a Cas9 nickase	Different base editors need different PAM	Independent of NHEJ and HDR; not induce DSBs	Fewer off‐targets than HDR	–	Only four possible edits (C/T, G/A, A/G, and T/C)	NA
	Prime editing	An engineered Cas9 (catalytically impaired Cas9 fused to a reverse transcriptase) and a pegRNA	5′‐NGG‐3′ PAM			All 12 possible base‐to‐base conversions	Difficult to deliver due to large molecule size	
	Transposon‐encoded CRISPR/Cas system	Tn7‐like transposase subunits and CRISPR effector	INTEGRATE: 5′‐CC‐3′ PAM; CAST: 5′‐GTN‐3′ PAM			Be able to apply for nonmitotic cells	Need more studies on human genome editing	

Abbreviations: ZFNs, Zinc finger nucleases; TALEN, transcription activator‐like effector nuclease; PAM, protospacer adjacent motif; NHEJ, non‐homologous end joining; HDR, homology‐directed repair; CRISPR, clustered regularly interspaced short palindromic repeats; DSB, double strand breaks.

## CONFLICT OF INTEREST

The authors declare that they have no conflicts of interests.
